# Excerpts

**Published:** 1890-05

**Authors:** John A. Miller

**Affiliations:** Prof. of Medical Chemistry and Toxicology at the Medical Department of the Niagara University


### Selections.

### EXCERPTS.

By JOHN A. MILLER, M. Sc., Ph. D.

Prof. of Medical Chemistry and Toxicology at the Medical Department of the Niagara University.

BACTERIUM LACTIS AEROGEUS: By Adolf Baginsky, (*Zeitsch. f. Physiol. Chem.*, xii., 434; xiii., 352. *Ber. Deut .Chem. Ges.*, xxiii., 27). According to the author this bacterium converts lactose (milk sugar) into acetic acid and acetone; the latter in small quantities. The former is further decomposed into carbon dioxide, hydrogen and methane. Neutral lactic acid salts are converted into butyric acid. This bacterium acts upon amylum only in the presence of oxygen, acetic acid being produced. The presence of sugar, as an intermediate product, could not be identified.

ANTHRAROBIN AND CHRYSAROBIN: By Th. Weyl, (*Arch. f. d. ges. Physiol.*, xliii., 367. *Ber. Deut. Chem. Ges.*, xxiii., 29). According to the author anthrarobin administered to rabbits in doses of 0.8 grm. for each kilogram of body weight is without toxic effect. Anthrarobin is excreted principally through the kidneys and the greater portion of it is found unchanged in the urine. A small portion is converted into alizarine and found as such in the urine.

Chrysarobin administered to dogs in doses of 0.1 grm. per kilogram weight produced violent vomiting, diarrhea and albuminuria. This substance appears after administration, in the urine, for the greater part unchanged, together with some chrysophanic acid.

STUDIES ON THE APPEARANCE OF RENNET IN THE HUMAN STOMACH UNDER PATHOLOGICAL CONDITIONS. By E. G. Johnson, (*Zeitschr. für Klin. Med.*, xiv., 240; *Ber. Deut. Chem. Ges.*, xxiii., 29). The author found rennet in the secretion of the stomach of persons suffering from various diseases of that organ. Rennet was present during all periods of digestion. It was absent in one case of chlorosis, in several cases of fever, and entirely absent in cancer of the stomach. The author did not find it either in the urine of man or in the gastric secretion of dogs.

Absorption of Fat in the Intestine. By A. Geuenhage and Krohn, (*Bied. Centr.*, xviii., 617; *Jour. Chem. Soc.*, lviii., 183). The author showed some time ago that the epithelial cells of the intestine cut out of a frog, as well as those in the living organism, are capable of taking up drops of fat from the intestinal tube filled with fat or emulsion.

The experiments were made with frogs that had been starved for some time, so that the intestines should be free from food constituents. The substances employed were milk, olive oil, lanolin emulsion, and a solution (?) of the finest Chinese ink; this last being employed in order to ascertain whether the epithelium absorption was confined to fatty substances alone. The results of the experiments show that the assumption of a mechanical activity of the epithelium of the intestines in taking up of fat is inadmissable, inasmuch as only fat, and not even the finest grains of other substances, enter into the protoplasma of the border cells. It is also established that the intestinal epithelium of hibernating frogs forms a store-place for excess of fat, and will retain fatty substances enclosed in it with great tenacity.

Origin of Uric Acid in Mammals. By J. Horbaczewski, (*Monatsh.* 10, 624; *Jour. Chem. Soc.*, 58, 185). The author believing that uric acid might be built synthetically from acrylic acid and some nitrogenous compound, such as urea administered with sodium acrylate mixed with the food, to a strictly dieted subject. No increase of uric acid was detectable in the urine.

The author further found that if a slow stream of air is passed through mixtures of fresh spleenic juice and defibrinated blood kept at 37° to 40°, very considerable quantities of uric acid are formed. This result is not produced by the blood alone, and must be regarded as due to a function of the spleen, which, perhaps, under some circumstances brings about the degradation of the white corpuscle of the blood.

Examination of Capsula Suprarenalis. By F. Marinozuco, (*Atti. I. R. Acc. d. Lincei. Rudct.*, 1888, 835; *Ber. Deut. Chem. Ges.*, xxiii., 57). The aqueous extract of capsula suprarenalis is very poisonous in its action. The author has made some experiments for the purpose of ascertaining the nature of the toxic principle. His conclusions are that the toxic effect is produced by *neurin glycero-phosphate.*

---

A new eye and ear infirmary has been incorporated by Drs. Abbott, Grove and Hinkle, who have withdrawn from the Buffalo Eye and Ear Infirmary. Its location and the hours of service will be ascertained by a reference to our advertising pages.

